# Critical and courageous thinking in medical education: truth-telling, an antidote to tradition

**Published:** 2018-05-31

**Authors:** Marcel D’Eon

**Affiliations:** 1 University of Saskatchewan, Saskatchewan, Canada

The CMEJ welcomes articles that will help improve policies and practices, studies that have implications for medical education and can make a difference in the lives of our students and patients. We thank all the authors and researchers who worked so hard to bring the fruits of their hard intellectual labours into the public eye. We know that they are telling us the truth as they have come to understand it and thus are contributing to achieving our mission. Truth matters, as our national neighbours to the south find themselves debating. Truth-telling is foundational to a flourishing human society and to a field of study.

Truth, we believe, is a little light in an otherwise dark world. It allows us to see, to see better, and even to see differently. Generating the light and releasing it upon our world is in itself a noble activity. Helping people to see and to see differently is the purpose of research. Light and truth are instrumental and creative; they shape and generate change, we all hope, for the better. The truth, delivered courageously, can be an antidote to staid tradition. As a force in the world and in medical education, we need not just the truth but those who will courageously speak truth to tradition.

It seems that medical education is on the cutting edge of tradition.^*^ While tradition can help us solve problems quickly and easily and allow organizations and institutions to function effectively, it can also impede progress. Some activities, once valuable and worthwhile, even state of the art innovations at the time, may, prevent better and more effective practices from emerging. When an ineffective aspect of medical education is defended and fed by tradition there is greater need for critical and courageous thinking. Perhaps this is where intelligent strangers might be able to play an important role. They may notice what we who are immersed in tradition simply take much for granted and cannot see the imperfections; they may be able to show us the truth of who we are and where we are headed better than we can ourselves.

Truth-telling is a common theme in popular culture. Artists as varied as Aretha Franklin, John Lennon, and Olivia Newton John (and many more) have songs about various aspects and applications of honesty. Stories like Pinocchio, To Kill a Mockingbird, A Few Good Men, Jim Carey’s Liar, Liar and of course, many, many others feature truth telling. There are few social or psychological imperatives as central to human flourishing as truth-telling.

But there are perils in expressing what one believes to be the truth. Courage is not supplementary but central to the enterprise of truth-telling. Whistleblowers are not plentiful; their open wounds and unsightly scars discourage applicants for the job. Even recently, two who have criticized CBME^1^ were not universally treated as heroes of medical education. Some ideas and some research may need to be stated subtly, almost subversively. Peter, Paul, and Mary sang it well in their song, *I dig rock and roll music*: “But if I really say it, the radio won’t play it Unless I lay it between the lines.”

Courage in telling the truth means not just facing persecution and opposition but also being open to correction, having enough humility to allow room for someone to convince you of a different, better view. When we engage in argumentation (truth telling with conviction) we must, as I have written before,^2^ be open to the possibility that we are wrong or at the very least that we don’t have the whole truth and can learn from others. This is the way forward; this is how we move past the cutting edge of tradition.

I will offer a few examples from the CCME 2018 where we ought to exercise our critical and courageous thinking. First, I will highlight a stellar example I observed personally. At the Learner Forum at noon on Sunday, a medical student from the University of Calgary stated from among the standing room only audience at the back something like the following (pardon the poor paraphrase), “All this focus on programs to develop resilience takes the focus off the system. If we fixed the system then I wouldn’t have to take resilience training.” I apologized to her later for starting to clap before she was finished delivering her entire comment! Many others clapped in support. It took insight and great courage to tell the deans that they had work to do. But she’s right. The title of the lunchtime forum, after all, included the term “Upstream”. What she was expressing, courageously (since there is some degree of blaming the victim in this medical student wellness situation), was the upstream public health concept of prevention through environmental and/or systems change. If the water in the well is making people sick, let’s not only give antibiotics to the sick and water purifiers to add to our water bottles but find a clean source of water – and fast! And just because we can’t find pristine water does not mean that the task of finding better water should be disparaged. I hope her critical and courageous thinking will bring truth and light to this situation and help create change. We can all learn from her.

Now here are six examples of maybe a dozen I noticed at CCME 2018:

- Supported exercise sessions (yoga or running) were scheduled for 600am. While wellness was a stated goal of CCME you could not both sleep or exercise at CCME unless on your own time.
- An oral presentation reported that a simple simulation exercise with the best outcome data was going to be offered as an optional extra-curricular activity while the lecture (the least effective) was being retained as a sanctioned curricular activity. (OF-1. Joobin Sattar, Queen’s University: Evaluation of Ear Disease Knowledge and Otoscopy Skills Transfer to Real Patients: A Randomized Controlled Trial)
- A prominent speaker argued for the use of the term andragogy (methodology of teaching adults) over pedagogy (teaching practices generally) without acknowledging the serious and harmful errors of the discredited adult learning movement.^3^
- One set of oral presentations was grouped under the heading of “non-cognitive.” What is it about ethical judgments and communications that does not involve important and high levels of thought?^4^
- An oral presentation reported patient priorities for the professional conduct of interviews. Patients, the people we serve, wanted to be respected and treated like valuable human beings by ethical practitioners, and only in fourth place comes, what we who run the system seem to value the most, a medical expert. (OF-4. Patricia Gerber University of British Columbia: Patient Insights and Perspectives on Competency in Professionalism: Implications for Health Education Curricula)
- Finally, for those of us in the audience for the opening plenary, who noticed a clear example of medical malpractice in Margaret Trudeau’s life story? Would you have spoken the truth courageously to that psychiatrist if you had had the chance back then?

I especially want to note one article and hence a set of articles in this issue. Congratulations to Persad who found the courage to describe his situation in “The unmatched,” draw profound personal lessons, grow from the experience, and write for the rest of us to benefit. Thanks also to the Canadian Federation of Medical Students and the Association of Faculties of Medicine Canada for contributing to the discussion of this vitally important issue.

On the next few pages is the introduction to the rest of the articles that comprise this issue of the CMEJ. You will notice that there are several about global medical education, articles that for a variety of reasons were not included in the Special Issue or Supplement on this topic.

Loranger et al. from the University of Ottawa in “Smoking cessation counselling training in the pre-clerkship curriculum of Canadian medical schools: A national survey” used epidemiological data to highlight the importance of smoking cessation to the health of the population. While cigarette use is Canada’s leading cause of preventable disease, disability and death (likely to be complicated if not exacerbated by legal marijuana^†^) and the Medical Council of Canada requires that physicians be able to address tobacco-use, training in smoking cessation counselling, however, remains largely neglected in the curriculum of many Canadian medical schools. The authors found both substantial deficits and inconsistencies in the delivery of smoking cessation education and that medical students are ill prepared to engage patients in a discussion about smoking cessation.

In “A Canadian medical student profile: Implications for admission, professional identity formation and career choice,” Harris and McKay argue that personality is one of the key elements in professional identity formation and specialty choice. Over a 10-year period, medical students from 11 of Canada’s 17 medical schools took the online Keirsey Temperament Sorter-II. Using Chi Square analysis, they found that the distribution of personalities [Guardian, Idealist, Artisan, and Rational] for medical students differs from the distribution reported for the general Canadian population and that the distribution of personalities is similar for each Canadian medical school. Knowing the personalities of medical students could be important for medical schools in such areas as admissions, career counselling and professional identity formation.In “Use of portfolios for assessment of global health residents: Qualitative evaluation of design and implementation” Gibson et al. describe how the Global Health training program chose to use portfolios to assess how residents at the University of Calgary met their career goals. Using an online survey, interviews, and focus groups they gathered perceptions of the portfolio. They found that, overall, residents and faculty were supportive of the use of portfolios for summative assessment, noting the value of authentic and varied assessments and mentor interaction as positive attributes. These findings provide additional confidence in tools for individualized yet rigorous assessment Global Health.

In “Ethical globalization? Opportunities for decolonizing theoretical frameworks for internationalization in Canadian medical education,” Bhandal argues that members of the medical education community need training and tools to navigate internationalization and north-south disparities. Using decolonization as a theoretical framework, Bhandal notes analytical gaps in discussions of the role of medical education in the context of colonial, neoliberal, unjust Global North-South relations.

Global health education initiatives often favour trainee growth over benefits to host communities. In “Community engagement in global health education supports equity and advances local priorities: an eight year Ecuador-Canada partnership” by Misra et al., the authors describe a two-month global health elective for medical trainees at McGill University that focuses on community engagement and participatory research to provide mutually beneficial outcomes. Multiple community benefits have been realized.

Warde et al. highlight the impact of globalization on patient education in medical training and practice, and how the use of plain language communication can mitigate this impact in “Capability in caring: Plain language communication as a priority competency for medical professionals in a globalized world.” They claim that curriculum developers at all levels of medical training must consider how to integrate plain language communication into their existing training programs to enable providers to better meet the needs of an increasingly globalized patient population.

“Students Working Against Tobacco: A novel educational program to improve Canadian medical students’ tobacco counselling skills” by Lammers et al. describes how medical students at the University of Ottawa created Students Working Against Tobacco (SWAT), a program that provides opportunities to discuss tobacco use, smoking prevention and cessation with elementary-school students. Following their involvement in the SWAT program, medical students’ smoking cessation counselling knowledge and skills improved.

“Ethics in radiology: A case-based approach” by Stiles-Clarke and Clarke describes the implementation of a case-based approach to teaching and learning ethics for Canadian radiologic residents at two different institutions using two different methods within one of the institution. Generally the residents found value in the learning experiences. A problematic finding was that the opportunity to prepare for the case discussion ahead of time was helpful for some but not for others.

Tang et al. in “Physician workforce planning in Ontario must move from short-term reactivity to long-term proactivity” decry the “Boom and bust” cycles of alternating physician surplus and undersupply. They claim that we have increasing numbers of Canadian physicians unable to secure residency positions and ultimately serve their communities. As Ontario moves forward in redefining its physician workforce strategy, medical school and residency enrollment should be key considerations, rather than afterthoughts.

The images in this issue are particularly evocative. Natter reminds us that the madness of the on-call life style is incongruous with the medical profession. We all know there are other contradictions and we cannot wait for him to illustrate these as well. In the cover photograph, Maruyama powerfully captures her longing for and fulfillment in motherhood together with - not in opposition to or competition with - her call to medicine.

Enjoy and profit from these studies and commentaries as we seek to improve and strengthen medical education in Canada and in the world.

## Footnotes

* Used by Sarita Verma at one of the plenary sessions at CCME 2018.

† The Editor’s personal opinion.
